# PEDOT:PSS Conductivity Enhancement through Addition of the Surfactant Tween 80

**DOI:** 10.3390/polym14235072

**Published:** 2022-11-22

**Authors:** Joseph L. Carter, Catherine A. Kelly, Jean E. Marshall, Vicki Hammond, Vannessa Goodship, Mike J. Jenkins

**Affiliations:** 1 School of Metallurgy and Materials, University of Birmingham, Birmingham B15 2TT, UK; 2 Warwick Manufacturing Group, University of Warwick, Coventry CV4 7AL, UK

**Keywords:** PEDOT:PSS, Tween 80, sheet resistance, microstructural analysis, surface tension, polymer thin films, polymer blend films

## Abstract

Replacement of indium tin oxide with the intrinsically conducting polymer poly(3,4–ethylenedioxythiophene):poly(styrenesulfonate) (PEDOT:PSS) has been of significant interest in recent years as a result of lower processing and material costs. In addition, the inclusion of additives has been reported to further enhance the conductivity, rheology, and wettability of PEDOT:PSS. In this study, Tween 80 was shown to decrease the sheet resistance of PEDOT:PSS films from approximately 1000 to 76 Ω□^−1^ at a 2.67 wt% surfactant concentration. Through X-ray diffraction, Raman spectroscopy, and atomic force microscopy, it was shown that the surfactant caused phase separation and structural ordering of the PEDOT and PSS components, leading to this improvement in conductivity. Furthermore, Tween 80 altered the rheological properties and decreased the surface tension of PEDOT:PSS, making coating common commodity polymers, often used as flexible substrates, more viable.

## 1. Introduction

**Figure 1:**
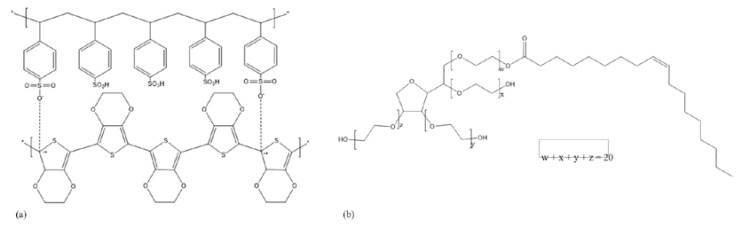
Chemical structures of (**a**) PEDOT:PSS and (**b**) Tween 80.

Poly(3,4–ethylenedioxythiophene):poly(styrenesulfonate) (PEDOT:PSS) (Figure 1a) is an intrinsically conducting polymer that has shown promise as an alternative to indium tin oxide (ITO) in organic optoelectric devices [1]. PEDOT:PSS has some major advantages over ITO, such as decreased material costs and superior mechanical flexibility, allowing it to be used on flexible polymer substrates [2,3]. PEDOT:PSS also has the potential to be applied to substrates using bulk manufacturing methods such as roll-to-roll (R2R) or inkjet printing (IJP), which allows for more accurate patterning, lower temperature requirements, and reduced running costs [4,5].

The main hinderance to the widespread use of pristine PEDOT:PSS is its relatively poor conductivity of approximately 1 S cm^−1^, and sheet resistance of 1800 Ω□^−1^ [6,7]. This is considerably inferior to ITO, with a conductivity of 3500 S cm^−1^ and sheet resistance of 20 Ω□^−1^ [8]. Improvements to PEDOT:PSS film conductivity using secondary enhancement mechanisms have already been largely reviewed in the literature [1,9,10]. To date, the most effective uses concentrated sulfuric acid as a post-treatment wash, generating a conductivity of 4300 S cm^−1^ [11]. However, this is not an ideal enhancement method due to the corrosive nature and negative environmental impacts of the acid. Furthermore, acid treatments could limit the applications of PEDOT:PSS by potentially making it unsuitable in bioelectronics as well as preventing bulk manufacture. Other organic compounds, such as ethylene glycol (EG) [12], dimethyl sulfoxide (DMSO) [13] and methanol [14], have been used as pre- and/or post-film formation treatment methods to enhance the conductivity of PEDOT:PSS films to approximately 735 [15], 898 [16], and 1360 [14] S cm^−1^, respectively (Table 1).

Surfactants have also been reported to enhance film conductivity when added to PEDOT:PSS solution prior to film formation (Table 1). The non-ionic surfactant, Triton X-100 (1 wt%), has been shown to enhance conductivity to 100 S cm^−1^ [20], with Tween 80 (also known as polysorbate 80), in combination with methyl ethyl ketone (MEK), also causing a significant drop in the sheet resistance of PEDOT:PSS films [21]. Other surfactants, such as anionic sodium dodecyl sulphate (SDS) and sodium dodecylbenzene sulfonate (SDBS), have been reported to enhance conductivity from 0.61 to 70 and 224 S cm^−1^, respectively [19]. It is thought that the addition of surfactants weakens the ionic interaction between PEDOT and PSS causing phase separation, which allows for greater alignment of the PEDOT chains through π–π stacking and a conformational change of the PEDOT backbone from benzoid to quinoid. The resulting more linear chain structure contains improved conductive pathways through PEDOT:PSS films, reducing the hopping distance of electrons between PEDOT-rich regions [20,22]. Additionally, surfactants alter the rheological properties, surface tension, and wettability of PEDOT:PSS solution, which are important considerations for bulk manufacturing processes [19,23,24].

Another important factor in the use of these formulations as electrodes is their transparency. Researchers have shown that the addition of EG, TDE, and glycerol to PEDOT:PSS solutions has no effect on the transparency of the resultant films [6,15] with transmissions ranging from 81 to 96% (Table 1).

Whilst the use of surfactants is better for the environment than strong acids, they have not yet enhanced PEDOT:PSS conductivity to the same level as ITO. However, work with non-ionic surfactants has been mostly focused on the use of the Triton X series. While Tween 80 has been studied [21,25], it has not been utilised alone, and often the focus is on improving wettability. 

In this study, the effect of solely adding the non-ionic surfactant Tween 80 (Figure 1b) to PEDOT:PSS solution as a conductivity-enhancing agent was investigated, utilising a broad range of characterisation techniques. The effects of Tween 80 on film quality, sheet resistance, conductivity, solution viscosity and wettability were investigated. Potential mechanisms for the observed conductivity enhancement were also probed via X-ray diffraction (XRD), Raman spectroscopy, and atomic force microscopy (AFM).

## 2. Materials and Methods

### 2.1. Materials

A high-conductivity, surfactant-free, aqueous dispersion of PEDOT:PSS (1.2 wt%) and polysorbate 80 (Tween 80) were obtained from Sigma-Aldrich (Gillingham, UK). All materials were used as received.

### 2.2. Solution Formation

PEDOT:PSS solutions containing a range of Tween 80 concentrations (0–3 wt%) were created in triplicate. All solutions were stirred with a magnetic stirrer for 10 min, to ensure sufficient mixing, and sonicated for 10 min, to break up any agglomerates.

### 2.3. Film Formation

Prior to casting, rectangular glass slides (1 × 2 cm) were washed with hot water and detergent, followed by acetone, before being rinsed with distilled water and dried. The glass substrates were dipped into the PEDOT:PSS/Tween 80 solutions, for 30 s, covering approximately half the slide. Resultant films were annealed at 140 °C for 1 h and left to equilibrate in ambient conditions for 12 h prior to testing.

### 2.4. Film Characterisation

#### 2.4.1. Sheet Resistance and Conductivity Measurements

Sheet resistance was measured using an Ossila 4-point probe (Ossila, Sheffield, UK) at a maximum of 1 V. Ten repeat measurements were taken at 6 locations across each film to remove any orientation bias in the samples. Film thickness was measured with an Ambios XP200 Stylus Profilometer (Ambios Technology, Santa Cruz, CA, USA). Five measurements were performed on each sample at a spacing of 1.5 mm and a scan length of 10 mm. Data was analysed using XP-Plus Stylus Profilometer software (Ambios Technology, Santa Cruz, CA, USA). Conductivity was calculated from sheet resistance using the following equation.

$$\sigma_{s}=\frac{1}{R_{s}\times t}$$

where $\sigma_{s}$ is the conductivity (S cm^−1^), $R_{s}$ is the sheet resistance (Ω□^−1^), and t is the film thickness (cm).

#### 2.4.2. XRD

XRD was performed using a 3rd generation Malvern Panalytical Empyrean XRD (Malvern, UK) equipped with multicore (iCore/dCore) optics and a Pixel3D detector operating in 1D scanning mode. Scans were made with Cu K_α1/2_ radiation (1.5419 Å) in the range 2*θ* =1.5–50 with a step size of 0.0263° and a count time of ~147 s/step.

#### 2.4.3. Raman

Raman spectroscopy was performed using a Renishaw inVia Raman microscope (Wotton-under-Edge, UK) at a wavelength of 532 nm. Spectra were normalised to the largest peak height to allow for comparison between samples.

#### 2.4.4. Atomic Force Microscopy

A NanoWizard II Atomic Force Microscope (AFM) (JPK, Berlin, Germany) was used in non-contact mode to measure the morphology of PEDOT:PSS/Tween 80 films with adhesion force mapping. A 50 × 50 µm area was measured to a resolution of 64 × 64 pixels. 

### 2.5. Solution Characterisation

#### 2.5.1. Rheology

A Netzsch Kinexus Pro+ Rheometer (Wolverhampton, UK), with a 4°, 40 mm cone, and plate geometry, was used to determine the viscosity of the solutions. Approximately 1.2 mL was pipetted onto the plate. Rotational tests were performed at 25 °C as the shear rate increased from 0.01–100 s^−1^.

#### 2.5.2. Surface Tension

The contact angle was measured by pipetting a single droplet of each solution onto a glass slide. An image was taken and the angle between the glass slide and the droplet analysed using ImageJ software (LOCI, University of Wisconsin, Madison, WI, USA). A capillary tube (Ø 0.8 mm) was then placed into the PEDOT:PSS/Tween 80 solutions and the maximum distance the liquid travelled up the capillary was measured. Surface tension was calculated using:

$$Surface\;tension=\frac{\rho gr}{2}\times \frac{h}{cos\theta }$$

where ρ is the solution density (kgm^−3^), g is the acceleration due to gravity (ms^−2^), r is the radius (m) of the capillary tube, h is the height (m) the solution travelled up the capillary tube, and θ is the measured contact angle (rad).

$$\rho =\frac{m_{PEDOT:PSS}+m_{Tween\;80}}{Vol_{PEDOT:PSS}+Vol_{Tween\;80}}$$

where the volumes of each component were calculated using the individual masses and densities. Density of PEDOT:PSS and Tween 80 were taken as 999 and 1064 kgm^−3^, respectively [26,27]. Two samples were analysed for the contact angle and capillary rise experiments, and the results were averaged prior to calculating surface tension. 

## 3. Results and Discussion

Good quality dip-cast films were successfully produced on the glass slides for each of the Tween 80 concentrations analysed. This was determined through visual analysis and was deemed to be ‘good’ if the film was free of defects, smooth, and displayed good adhesion to the substrate.

### 3.1. Sheet Resistance and Conductivity

**Figure 2:**
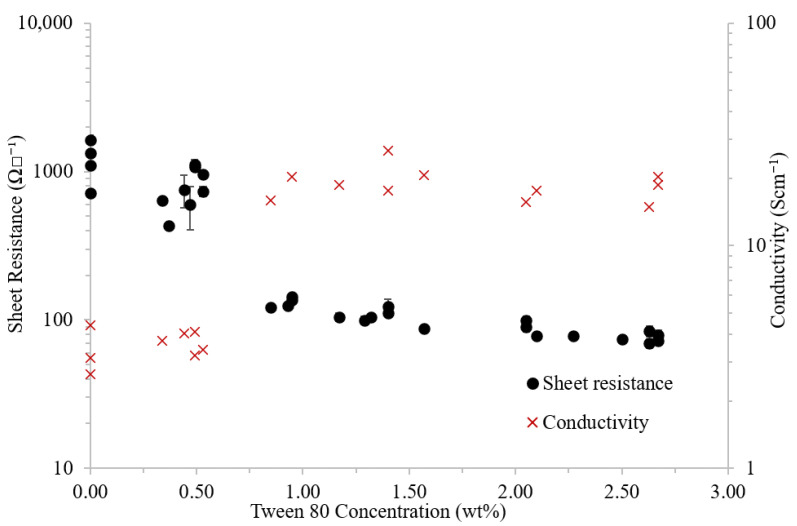
Sheet resistance (Ω□^−1^) (black circle) and corresponding conductivity (S cm^−1^) (red cross) of PEDOT:PSS films with various concentrations of Tween 80 (wt%). Error bars on sheet resistance show ± 1 standard deviation of 6 measurements across various locations on the film.

Pristine PEDOT:PSS films displayed a sheet resistance between 715 and 1640 Ω□^−1^ (Figure 2) (Table 2). The addition of a low concentration (approximately 0.5 wt%) of Tween 80 created a small reduction in the sheet resistance with a more severe drop observed as the concentration was increased to 0.9 wt%. Above 1 wt% surfactant, the sheet resistance continued to reduce with concentration; however, the effect diminished. 

As expected, conductivity showed the reverse trend with an increase from 3 to 20 Scm^−1^ being obtained at 0.9 wt% Tween 80 (Figure 2) (Table 2). Contrary to the sheet resistance results, above this concentration Tween 80 had little effect on conductivity, as the films were also found to increase in thickness. 

It has been suggested in the literature that surfactants will initially interact with the excess PSS in solution before disrupting the ionic bond between PEDOT and PSS [28]. Based on the sheet resistance and conductivity results (Figure 2) it is suspected that complete saturation of the excess PSS does not occur until between 0.5 and 0.8 wt%. Above this concentration, the Tween 80 will interfere with the bound PEDOT:PSS, resulting in a significant reduction in sheet resistance coupled with an increase in conductivity. 

Reductions in sheet resistance and/or increases in conductivity have been observed for a range of additives such as PEG of various molecular weights [12], SDS [19], SDBS [19], the Triton-X series [28], 2,2-thiodiethanol [6], and glycerol [6]. As seen with Tween 80, in the majority of these cases, a minimum concentration of additive is required prior to any significant change being observed. Similar plateaus in electrical performance are reported in the literature, however, the concentration at which this occurs depends on the additive [6,12,28].

### 3.2. Microstructural Analysis

A range of analyses were performed to probe the increase in conductivity observed on the addition of Tween 80.

#### 3.2.1. XRD

**Figure 3:**
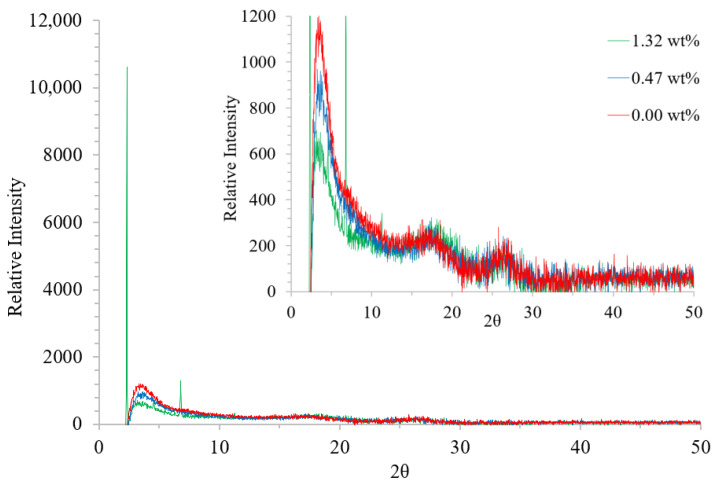
XRD traces showing the relative intensity of diffraction peaks for differing films. From bottom to top, traces are as follows: pristine PEDOT:PSS (red) and PEDOT:PSS/Tween 80 with 0.47 (blue) and 1.32 (green) wt% surfactant. Insert displays a close up of the smaller peaks.

The X-ray diffraction pattern of pristine PEDOT:PSS displayed three distinct peaks at 2*θ* values of approximately 3.4, 17.4, and 26.9° (Figure 3). As calculated using Bragg’s Law, 2*d* sin*θ* = *λ*, these values correspond to lattice *d* spacings of 25.8, 5.1 and 3.3 Å, respectively. The *d* spacing of 25.8 Å (2*θ* = 3.4°) has been attributed to the distance between the lamella stacking (*d*_100_) of alternating PEDOT and PSS chains [16]. The amorphous halo of PSS was observed at a *d* spacing of 5.1 Å (2*θ* = 17.4°) whereas, the peak at 2*θ* = 26.9°, giving a *d* spacing of 3.3 Å, corresponded to the distance between the π-π stacking (*d*_010_) of PEDOT chains [11].

With the exception of a reduction in the intensity of the peak at approximately 2*θ* = 3.4°, indicative of a loss of order, no changes were observed to the diffraction pattern on the addition of 0.47% Tween 80. However, deviations in the sizes and positions of the peaks were observed with the higher concentration. The peak at approximately 2*θ* = 3.4° reduced in both intensity and angle, resulting in an increase in the *d* spacing. These factors demonstrate an increased separation between the PEDOT:PSS lamella and a reduction in crystallinity of this particular ordering [16]. In addition, extra peaks were observed at 2.33, 6.79, and 11.28°, corresponding to *d* spacings of 37.9, 13.0, and 7.8 Å, respectively. Similar peaks have previously been observed when PEDOT:PSS has undergone acid washes with sulfuric acid and were attributed to the lamella stacking of distinct additional PEDOT:PSS orderings with high crystallinities [11]. No change was observed to the peak at approximately 2*θ* = 26.9°, and therefore, the π-π stacking of the PEDOT chains.

#### 3.2.2. Raman

**Figure 4:**
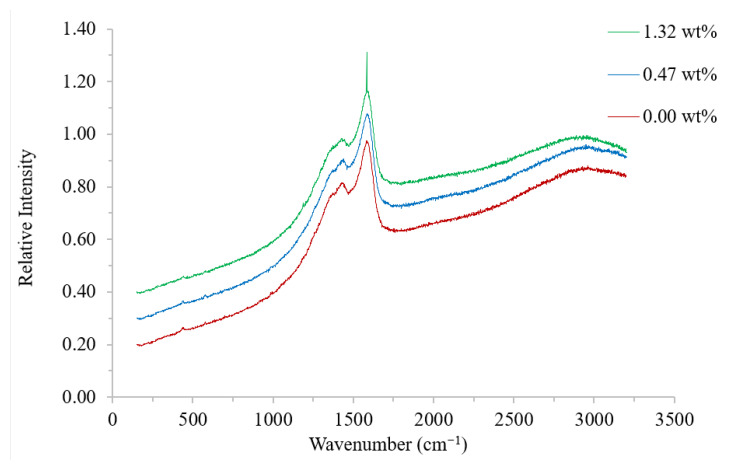
Raman spectra of PEDOT:PSS/Tween 80 films containing 0.00 (red), 0.47 (blue), and 1.32 (green) wt% surfactant (from bottom to top).

Raman analysis was performed on the PEDOT:PSS/Tween 80 films to probe for possible changes to the PEDOT double-bond structure from benzoid to quinoid. Pristine PEDOT:PSS displays three main peaks in the Raman spectra (Figure 4). The peaks at approximately 1372 and 1429 cm^−1^ are attributed to the C_β_ - C_β_ and C_α_ = C_β_ stretching vibrations, respectively, within PEDOT, whereas, the peak at approximately 1586 cm^−1^ was created by the PSS component [29]. Although Tween 80 is Raman active, it has a much lower intensity compared to PEDOT:PSS, with no difference observed between the three traces in the region of the expected main peak (approximately 2800 cm^−1^) [30].

Within the literature, it has been reported that the benzoid to quinoid resonance structural change can be seen through a red shift and narrowing of the band at approximately 1429 cm^−1^, representing the C_α_ = C_β_ bond in the thiophene ring of PEDOT [22,31]. These shifts have been observed with both the non-ionic surfactant Triton X-100 [20] and the organic solvent ethylene glycol [31]. Alternatively, Chang et al. reported changes to the ratio of the peak heights at 1372 (C_β_ - C_β_) and 1429 (C_α_ = C_β_) being indicative of the benzoid to quinoid shift when a 2:1 ethylene glycol:hexafluroisopropylalcohol mixture was used as a post-processing wash [29]. Neither of these changes were observed in the present study, suggesting that this resonance structural change does not occur with the surfactant Tween 80. The surfactants SDS and SDBS have also been reported to display no changes to the Raman spectra of pristine PEDOT:PSS, and in these cases the enhanced conductivity was attributed to an increase in the size of the conducting domains [19]. 

#### 3.2.3. AFM

**Figure 5:**
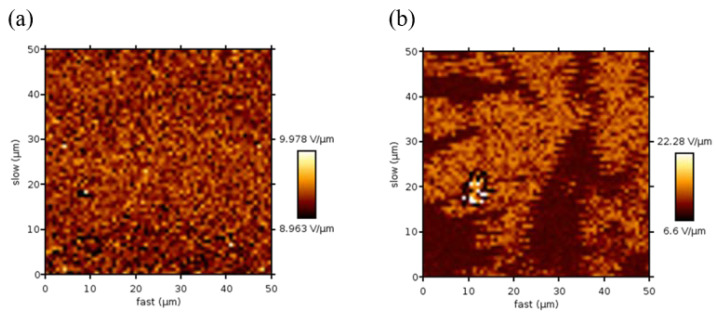
Topographic AFM images showing the adhesion scan normalised by the surface profile of (**a**) pristine PEDOT:PSS and (**b**) PEDOT:PSS films containing 1.40 wt% Tween 80.

AFM analysis of pristine PEDOT:PSS film shows an even distribution of both polymers throughout the surface (Figure 5a). However, in the presence of 1.40 wt% Tween 80 there is a clear degree of phase separation (Figure 5b), with the large dark patches believed to be the surfactant. Kim et al. previously demonstrated a similar phase separation when high concentrations of Triton X were added to PEDOT:PSS spin-coated films [28]. The authors observed that as the concentration of Triton X was increased there was an obvious distinction where immiscibility occurred dependent on the molecular weight of the surfactant. This immiscibility was also coupled with a sharp decrease in sheet resistance comparable to that seen here with Tween 80. Kim et al. postulated that at low concentrations, the surfactant only interacts with the excess PSS [28]. As the concentration is increased further towards the miscibility boundary, the surfactant interacts with the PEDOT and PSS, leading to a reduction in sheet resistance. Above the saturation point, the excess surfactant is phase-separated and the sheet resistance begins to plateau. Based on the results seen here and the similarity of the two surfactants, it is likely that the same trend occurs with Tween 80.

Numerous theories on the effect of additives, in particular surfactants, on the sheet resistance and/or conductivity of PEDOT:PSS films have been proposed in the literature [12,17,19,20,22,28,32,33,34,35]. The most widely publicised are: screening of PEDOT and PSS, phase separation of both polymers, formation of nanofibrils, conformational changes to the structure of PEDOT, and combinations of these effects. These factors are all reported to reduce the barriers to electron flow and, therefore, improve conductivity by creating better conducting pathways. 

Sorbitol, PEG, Triton-X, and the polar solvents DMSO, dimethylformamide (DMF), and tetrahydrofuran (THF) have all been reported to produce a screening effect, weakening the Coulombic interaction between PEDOT and PSS [12,22,28,33,34,35]. In some cases, this was caused by the ability of the additives to penetrate the PEDOT–PSS bond [22,28,34]; whereas in others, exterior interactions caused a weakening of this bond [12]. In the present work, the increase in PEDOT:PSS lamella separation shown by XRD could be an indication of this screening effect. 

The screening effects allow phase separation to occur, creating PEDOT islands within a PSS matrix [20,22,28,33]. These islands have been described using AFM analysis as clusters, elliptical grains, and nanofibrils. The growth of these domains with increasing additive concentration leads to better conducting pathways and, therefore, an increase in conductivity [12,19]. AFM analysis following the addition of Tween 80 shows phase separation on a much larger scale and is likely to be excess surfactant [28]; however, the increased order observed by XRD may be generated through the formation of PEDOT nanofibrils or elliptical grains. 

Authors have also reported a conformational change to the PEDOT structure [17,20,22,32]. In the natural state, the short chains of PEDOT are bound to the longer PSS chains. This results in the formation of coils as the PSS chains repel each other. Phase separation of the two polymers allows PEDOT to realign into an extended coil or linear structure, resulting in a change from a benzoid to quinoid structure. Raman spectroscopy has been used to display this change when diethylene glycol and Triton X-100 have been added to PEDOT:PSS [17,20,22,32]. However, others have reported no conformational changes with polar solvents, SDS, or SDBS using the same technique [19,34].

In the present study, no conformational change was observed by Raman; however, increased ordering was found by XRD. These results suggest that phase separation occurs, leading to the formation of more ordered PEDOT segments within a PSS matrix; however, the effect is not severe enough to cause a conformational change to the PEDOT structure. The formation of these PEDOT islands improved the conducting pathways through the film, causing the reduction in sheet resistance observed. These results also show that a minimum Tween 80 concentration is required to produce an effective response, with no change to the XRD pattern or sheet resistance found below 0.9 wt%.

### 3.3. Solution Analysis

In addition to improving the conductivity of PEDOT:PSS, surfactants can also alter the solution properties. This can impact the viscosity and wettability, which in turn has implications for bulk manufacturing and adhesion to substrates. 

#### 3.3.1. Rheology

**Figure 6:**
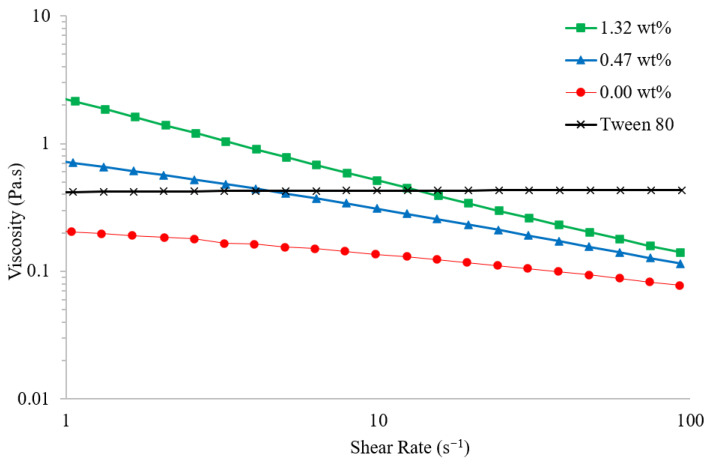
Shear viscosity (Pa.s) of pristine PEDOT:PSS/Tween 80 solutions with surfactant concentration 0.00 (red circle), 0.47 (blue triangle), and 1.32 wt% (green square); and pure Tween 80 (black cross) for varying shear rates (s^−1^).

The rheology of a range of PEDOT:PSS/Tween 80 formulations was assessed to determine the suitability for processing via bulk manufacturing methods such as roll-to-roll and inkjet printing. Pristine PEDOT:PSS is a non-Newtonian fluid displaying a continuous decrease in viscosity with increasing shear rate (Figure 6). The viscosity of Tween 80 stays relatively constant regardless of the shear rate, and is greater than pristine PEDOT:PSS throughout. Despite the Newtonian behaviour of Tween 80, solutions of PEDOT:PSS containing the surfactant were also found to shear thin (Figure 6). Hoath et al., previously reported similar non-Newtonian behaviour when PEDOT:PSS is mixed with the surfactants Dynol 607 and Zonyl F50-100 [24].

**Figure 7:**
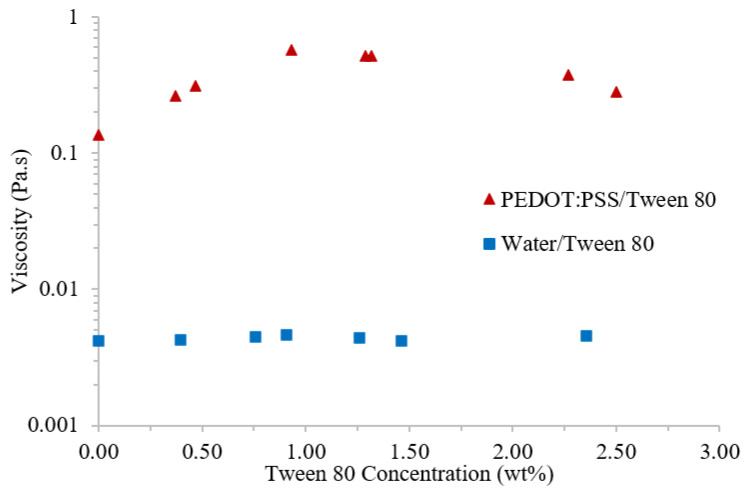
Viscosity (Pa.s) at a shear rate of 10 s^−1^ of PEDOT:PSS/Tween 80 (red triangle) and water/Tween 80 (blue square) solutions, with varying Tween 80 concentration (wt%).

On the addition of small amounts (<1 wt%) of Tween 80, the viscosity of PEDOT:PSS was found to increase with surfactant concentration (Figure 7) (Table 3). This is in agreement with literature reports for the addition of the surfactants Dynol 607 and Zonyl F50-100 [24]. Above this concentration, a plateau was observed until the viscosity begins to decrease again above 2 wt% Tween 80. As PEDOT:PSS solution contains mainly water, the effect of the same Tween 80 concentrations on pure water were evaluated. These tests revealed a constant viscosity throughout (Figure 7), highlighting that the changes observed were a result of interactions between the surfactant and PEDOT:PSS.

To the authors’ knowledge, no literature has been published to date on the rheology of PEDOT:PSS with surfactants or other additives at these higher concentrations. One possible explanation for the trend in viscosity is centered on the concept of which components the Tween 80 interacts with. Initially, Tween 80 is believed to interact only with the excess PSS in solution [28]. Based on the sheet resistance results (Figure 2), the saturation of the excess PSS by Tween 80 is expected to occur below 0.5 wt% surfactant. Above this concentration, the Tween 80 begins to disrupt the bond between the PEDOT and PSS components. It has previously been suggested that the interaction of surfactants with PEDOT:PSS results in the creation of PEDOT-surfactant and PSS-surfactant complexes [22]; therefore, the Tween 80 will be contributing to the overall polymer content in the dispersion, increasing viscosity. Following this interaction, any excess Tween 80 has been reported to phase-separate and will begin to dilute the solution, reducing its viscosity towards that of pristine PEDOT:PSS.

These results also inform the suitability of PEDOT:PSS/Tween 80 solutions for bulk manufacturing. Tween 80 has previously been shown to be an effective additive to PEDOT:PSS for IJP and R2R methods when used in conjunction with other additives [36,37]. IJP and R2R generally favour lower viscosity inks. While limitations to attainable shear rates make it difficult to determine solution suitability for bulk manufacturing, pristine PEDOT:PSS and PEDOT:PSS containing 1% Tween 80 are thought to be appropriate [24,25]. As the viscosities of these two concentrations were found to be the highest and lowest in the current study, it suggests that any of the PEDOT:PSS/Tween 80 formulations analysed would be suitable for bulk manufacture. 

#### 3.3.2. Surface Tension

**Figure 8:**
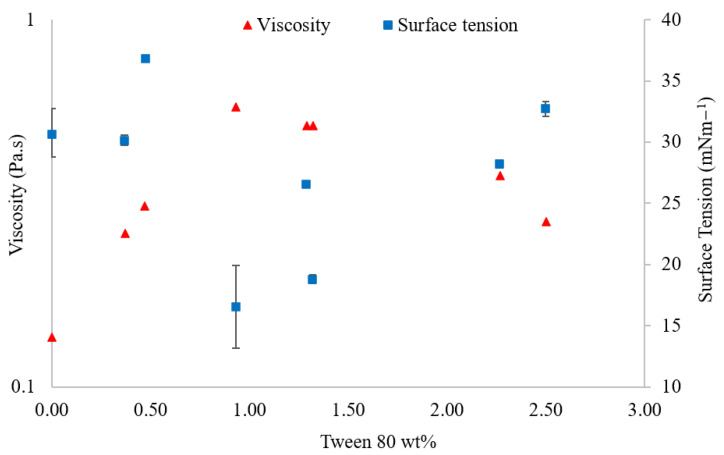
Viscosity (Pa.s) (red triangle) and surface tension (mNm^−1^) (blue square) variation with differing Tween 80 concentrations. For each formulation, two measurements were recorded for the contact angle and capillary height used to calculate the surface tension. The error bars represent the lowest and highest surface tensions calculated from these results to generate an impression of the error of the measurement technique.

The surface tension of PEDOT:PSS/Tween 80 solutions with varying surfactant concentrations was assessed. With the addition of Tween 80, surface tension showed an inverse relationship to viscosity (Figure 8) (Table 3), with 0.93 wt% Tween 80 causing both the lowest surface tension and the greatest viscosity. 

A drop in surface tension was seen with alternative surfactants, although in most cases only a single concentration was analysed [18,23,24]. Triton X-100 shows a progressive reduction in surface tension to 1 wt%; however, a plateau rather than an increase was observed as the concentration exceeded this point [20].

A lowering of the surface tension is advantageous when considering bulk manufacturing, as a lower surface tension will improve the wettability of the solution, allowing for a more even coating. This is particularly important when considering polypropylene, polyethylene terephthalate (PET), or other polymeric substrates, since pristine PEDOT:PSS solution coverage and adhesion on these substrates are usually poor [19]. By lowering the surface energy, and more closely matching this between solution and substrate, higher quality films can be produced. 

## 4. Conclusions

Tween 80 has previously been used as an additive to improve the wettability of PEDOT:PSS for the gravure printing of organic light emitting diodes; however, to date, its potential as a conductivity-enhancing agent has not been probed. This is despite other surfactants showing promise. Herein, it has been shown for the first time that Tween 80 can act as an effective additive to reduce the sheet resistance and increase the conductivity of PEDOT:PSS films, while also modifying the solution properties to facilitate bulk processing. Although it may initially appear that Tween 80 does not show as great an improvement as some other surfactants, such as Triton X-100, these results cannot be directly compared, as the films reported in the literature have also undergone post-treatment with solvent washes, which is known to enhance conductivity significantly. Therefore, if the current formulations were also washed, a greater improvement would be expected. 

The addition of small quantities of Tween 80 was shown to reduce the sheet resistance and improve the conductivity of PEDOT:PSS films, with a more significant change observed at 0.9 wt% surfactant. Above this concentration, only a small further reduction in sheet resistance was observed, while the conductivity plateaued. AFM and XRD analysis suggest the occurrence of phase separation leading to more ordered PEDOT segments within the PSS matrix. However, this is not significant enough to produce benzoid to quinoid conformation changes in the Raman spectra. The formation of these PEDOT islands leads to better conducting pathways and a reduction in sheet resistance.

Furthermore, the rheology and surface tension of PEDOT:PSS were modified by the addition of Tween 80. The reduced surface tension observed between 0.9 and 1.5 wt% surfactant implies enhanced wettability and is a good indication that these formulations would produce better films on flexible polymer substrates, which would otherwise coat poorly with pristine PEDOT:PSS.

## Figures and Tables

**Table 1:** Summary of effective secondary conductivity-enhancing agents reported to date.

Additive	Concentration (%)	Conductivity Change (S cm^−1^)	Transparency	Ref.
2,2′-thiodiethanol (TDE)	5	15 to 98	84%	[6]
Diethylene glycol (DEG)	0.3	0.006 to 10	Yes	[17]
DMSO	5	0.69 to 898	NR	[16]
	10	2.5 to 1233	NR	[18]
Dodecylbenzene sulphonic acid (DBSA)	2	1 to 500	NR	[13]
EG	6	0.3 to 640	~93%	[12]
	6	1 to 735	No change	[15]
Glycerol	5	15 to 57	81%	[6]
	6	0.782 to 152	NR	[5]
N-methyl-2-pyrrolidone (NMP)	20	0.03 to 30	NR	[7]
PEG (Mw 200, 300 and 400)	2	0.3 to 805	93%	[12]
Sodium dodecylbenzene sulphate (SDBS)	10	0.61 to 224	NR	[19]
Sodium dodecyl sulphate (SDS)	10	0.61 to 70	NR	[19]
Triton X-100	1	0.24 to 100	96%	[20]

NR: Not reported.

**Table 2:** Summary of the parameters obtained for each of the PEDOT:PSS/Tween 80 films.

Tween 80 (wt%)	Thickness (µm)	Sheet Resistance (Ω□^−1^)	Conductivity (S cm^−1^)
0.00	2.31	1639.49	2.64
0.00	2.08	1096.41	4.39
0.00	2.40	1334.68	3.12
0.00	-	715.67	-
0.34	4.18	637.85	3.75
0.37	-	429.53	-
0.44	3.28	757.14	4.02
0.47	-	597.84	-
0.49	2.27	1074.19	4.11
0.49	2.78	1120.75	3.21
0.53	3.08	955.35	3.39
0.53	-	736.25	-
0.85	5.11	121.96	16.04
0.93	-	125.08	-
0.95	3.43	142.38	20.46
0.95	-	136.58	-
1.17	5.11	104.47	18.75
1.29	-	99.57	-
1.32	-	104.37	-
1.40	3.34	111.84	26.80
1.40	4.57	123.75	17.68
1.57	5.53	87.15	20.75
2.05	6.42	98.72	15.77
2.05	-	89.11	-
2.10	7.25	77.97	17.69
2.27	-	78.40	-
2.50	-	74.24	-
2.63	8.00	83.73	14.93
2.63	-	69.36	-
2.67	6.70	79.29	18.84
2.67	6.77	72.33	20.41

**Table 3:** Summary of the viscosity and surface tension obtained for each of the PEDOT:PSS/Tween 80 solutions.

Tween 80 (wt%)	Viscosity (Pa.s)	Surface Tension (mNm^−1^)
0	0.136	30.61
0.37	0.262	30.12
0.47	0.310	36.82
0.93	0.579	16.52
1.29	0.515	26.57
1.32	0.514	18.81
2.27	0.376	28.22
2.50	0.281	32.72

## Data Availability

Available on request.
